# Effects of 8-week polarized vs. threshold training on cardiorespiratory fitness, post-exercise heart-rate recovery, and target accuracy under fatigue in table tennis players

**DOI:** 10.3389/fphys.2026.1926017

**Published:** 2026-08-19

**Authors:** Wenyuan Wang, Yushan He, Enli Xie

**Affiliations:** 1 School of Physical Education, Shanghai Normal University, Shanghai, China; 2 School of Sports Training, Nanjing Sport Institute, Nanjing, China

**Keywords:** autonomic recovery, polarized training, table tennis, technical stability, ventilatory threshold

## Abstract

**Background:**

Table tennis training requires appropriate intensity distributions to improve physical conditioning without impairing skill execution. This study compared the effects of an 8-week polarized training (POL) model with those of a threshold training (THR) protocol in table tennis players.

**Methods:**

Thirty-two male table tennis players (Tier 2) were randomly allocated (1:1) to either the POL intervention (80% Zone 1, 5% Zone 2, and 15% Zone 3) or the THR intervention (50% Zone 1, 45% Zone 2, and 5% Zone 3). Participants completed five supervised on-court training sessions per week, with internal workload monitored and targeted to be similar between groups. Cardiopulmonary exercise testing was used to quantify maximal oxygen uptake ( $\dot{V}O_{2\max}$) and ventilatory thresholds (VT1 and VT2). An incremental topspin protocol was used to assess sport-specific time to exhaustion (TTE), target accuracy during the terminal stage of the incremental protocol, and heart rate recovery at 60 s (HRR60).

**Results:**

The POL intervention produced greater physiological and performance adaptations than the THR intervention. VT1 increased to a greater extent in the POL group than in the THR group (+9.8% vs. +6.4%, *p* = 0.03). VT2 improved only in the POL group (+5.0% vs. −1.0%, *p* < 0.001). The POL protocol also increased sport-specific TTE (+2.7%, *p* = 0.049), improved target accuracy under fatigue (+7.8%, *p* = 0.041), and enhanced HRR60 (+11.7%, *p* = 0.040). The THR group showed no statistically significant improvements in these outcomes. No significant group-by-time interaction was observed for absolute $\dot{V}O_{2\max}$ (*p* = 0.821).

**Conclusion:**

This study suggests that an 8-week polarized training program may be more effective than threshold training for improving submaximal cardiorespiratory fitness, sport-specific endurance, fatigue-induced technical stability, and autonomic recovery in competitive male table tennis players.

## 1 Introduction

Table tennis requires a highly specific physiological profile. Players perform repeated explosive rallies interspersed with brief recovery periods (Pion et al., 2015). Anaerobic power contributes to stroke execution and depends on efficient energy transfer through the lower-limb kinetic chain (He et al., 2021). These anaerobic demands are supported by an aerobic base, which influences phosphocreatine resynthesis and lactate clearance between points (Tomlin and Wenger, 2001). Cumulative fatigue can alter joint kinematics and delay muscle activation as matches progress (Martin et al., 2016). These neuromuscular impairments may compromise stroke precision and tactical execution. Therefore, the ability to sustain high-quality stroke mechanics across multiple games may depend, in part, on targeted cardiorespiratory fitness (CRF) conditioning.

Aerobic conditioning in racket sports has traditionally followed a threshold-based (THR) model (Wenger and Bell, 1986). In this model, coaches allocate a substantial proportion of training volume to intensities at or near the second ventilatory threshold (Zone 2). Although continuous endurance athletes often use this zone to develop foundational aerobic capacity, skill-dominant athletes may experience distinct physiological costs from chronic Zone 2 exposure. Moderate-intensity work may induce residual neuromuscular fatigue without consistently providing a maximal aerobic stimulus. This accumulated load may prolong sympathetic nervous system activation (Kjøsen Talsnes et al., 2024). Such residual fatigue may be particularly relevant to table tennis, where stroke execution depends on precise multijoint coordination across the kinetic chain (He et al., 2021). Previous research has shown that physical fatigue alters coordination strategies during the table tennis forehand stroke, requiring compensatory movement adjustments to maintain accuracy (Aune et al., 2008).

Polarized training (POL) redistributes training intensity to reduce these physiological and autonomic burdens (Foster et al., 2022). This framework assigns approximately 80% of training volume to low intensity exercise (Zone 1, below VT1) and 15–20% to high intensity exercise (Zone 3, above VT2). It intentionally minimizes exposure to Zone 2 training (Stöggl and Sperlich, 2014). Evidence from endurance populations suggests that POL is associated with improvements in $\dot{V}O_{2\max}$, velocity at lactate threshold, and fractional utilization compared with THR models (Filipas et al., 2022; Nøst et al., 2024; Silva Oliveira et al., 2024). These adaptations are relevant to the physiological demands of racket sports. High-volume low-intensity training may enhance parasympathetic modulation (Buchheit, 2014). Improved systemic recovery may also reduce excessive mechanical and metabolic stress. In contrast, discrete high-intensity intervals may provide a strong central cardiovascular stimulus and recruit fast-twitch fibers required for multidirectional movement (Rivera-Köfler et al., 2025).

However, empirical evidence on POL in intermittent, skill-based sports remains limited. Table tennis requires athletes to clear metabolic byproducts and restore autonomic balance within short recovery intervals. Post-exercise heart rate recovery (HRR) is a practical marker of rapid parasympathetic reactivation (Stanley et al., 2013). Athletes with faster HRR may be better able to maintain technical execution under systemic fatigue (Daanen et al., 2012). By restricting moderate-intensity exposure, POL may create a physiological environment that supports rapid autonomic recovery. Preserving upper- and lower-limb biomechanics during late-stage match scenarios may therefore depend partly on this recovery pathway.

This randomized controlled trial compared an 8-week sport-specific POL program with a THR protocol in male table tennis players. We assessed sport-specific time to exhaustion (TTE), target accuracy under fatigue, and heart rate recovery at 60 s (HRR60) as primary outcomes, and markers of cardiorespiratory fitness ( $\dot{V}O_{2\max}$, VT1, VT2) as secondary outcomes. We hypothesized that the POL group would show greater improvements in autonomic recovery and higher target accuracy under fatigue than the THR group, with both models producing comparable improvements in $\dot{V}O_{2\max}$.

## 2 Methods

### 2.1 Study design

This parallel-group RCT compared an 8-week POL intervention with a THR model in table tennis players. The primary outcomes was sport-specific time to exhaustion (TTE), based on the sample-size calculation. Target accuracy under fatigue and heart rate recovery at 60 s (HRR60) were secondary outcomes. Cardiorespiratory fitness markers ( $\dot{V}O_{2\max}$, VT1, VT2, peak heart rate) were additional secondary outcomes. The study protocol was approved by the Institutional Review Board of Shanghai Normal University for Human Subjects (Approval No. 2026-058) and was conducted in accordance with the Declaration of Helsinki. All participants provided written informed consent before enrollment.

### 2.2 Participants

The sample size was estimated *a priori* using G*Power software (version 3.1; Heinrich Heine University Düsseldorf, Düsseldorf, Germany). Because specific evidence on POL in table tennis is limited, the calculation was based on the primary outcome (TTE) and assumed a moderate group × time interaction effect (Cohen’s f = 0.25), consistent with interaction effects reported in comparable short-term training studies (Stöggl and Sperlich, 2014), with α = 0.05 and 1-β = 0.80. A total of 28 participants was required; 32 were recruited to account for potential attrition (≈15%). Thirty-two male table tennis players were recruited from regional clubs and university teams by a research assistant who was not involved in outcome assessment. The participants were 21.9 ± 2.6 years old, 174.5 ± 5.2 cm tall, and had a body mass of 68.4 ± 6.8 kg, a BMI of 22.4 ± 1.7 kg/m^2^, and 8.2 ± 3.1 years of training experience. They met the McKay et al. (2022) criteria for Level 2 trained/developmental athletes (McKay et al., 2022). Eligible participants were adults who had completed at least three years of systematic training and had maintained a minimum of four training sessions per week during the previous three months. Before enrollment, they completed an average of 11.3 ± 1.6 h of table tennis training per week. Individuals were excluded if they had recent cardiometabolic or respiratory disease, current musculoskeletal injury, concurrent enrollment in another experimental study, or use of medications known to affect autonomic nervous system function.

### 2.3 Procedures and test methods

Baseline assessments began with anthropometric measurements and a familiarization session with the testing equipment. Participants were instructed to maintain their usual dietary habits, abstain from alcohol and caffeine for 24 h before testing, and avoid strenuous physical activity for 48 h before each assessment. Baseline testing was conducted on separate days to minimize residual fatigue and followed a standardized sequence: cardiopulmonary exercise testing (CPET), followed by sport-specific technical stability and recovery protocols. The same testing order and environmental conditions were replicated during the post-intervention assessments.

After baseline data collection, an independent statistician used a computer-generated randomization sequence to allocate participants to either the POL or THR group. Allocation concealment was ensured using sequentially numbered, opaque, sealed envelopes, which were opened by a research assistant who then informed the coaches of group assignments. Participants and coaches could not be blinded to group allocation given the nature of the intervention. Assessors responsible for physiological and performance evaluations, and the data analyst, remained blinded to group allocation throughout the 8-week supervised intervention and post-intervention testing.

#### 2.3.1 Cardiorespiratory fitness assessment

Maximal oxygen uptake ( $\dot{V}O_{2\max}$) was determined using treadmill-based CPET. Participants completed a 3-min warm-up at 5.0 km·h^−1^. The protocol then increased the speed to 8.0 km·h^−1^, after which the gradient was increased by 2% every 2 min. Testing continued until volitional exhaustion or until participants were unable to maintain the required workload. Breath-by-breath respiratory gases were collected using a portable metabolic system (CORTEX Biophysik GmbH, Leipzig, Germany). $\dot{V}O_{2\max}$ was defined as the highest 30-s average $\dot{V}O_{2\max}$ value recorded during the test. Maximal exertion was confirmed by volitional exhaustion together with predefined physiological criteria. These criteria included a respiratory exchange ratio of ≥1.15, a heart rate of ≥95% of the age-predicted maximum, and an inability to continue despite standardized encouragement (Midgley et al., 2007). If a definitive $\dot{V}O_{2\max}$ plateau, defined as an increase of<150 mL·min^-1^ or<2 mL·kg^-1^·min^-1^ between the final stages, was not observed, the value was reported as $\dot{V}O_{2\max}$ peak (Poole et al., 2008). Two blinded investigators independently identified the first and second ventilatory thresholds (VT1 and VT2) using the V-slope method and ventilatory equivalents method (Beaver et al., 1986). All CPET sessions were scheduled within a fixed afternoon time window to control for diurnal variation. Testing times were matched between the baseline and post-intervention assessments.

#### 2.3.2 Maximal incremental test

Sport-specific performance was assessed using a table tennis-specific incremental exhaustion test performance (Zagatto et al., 2011). A programmable ball-feeding robot delivered topspin balls to a fixed forehand location. Participants performed forehand topspin strokes at an initial frequency of 70 balls·min^-1^. Ball frequency was increased by 5 balls·min^-1^ every 2 min. The test was terminated at volitional exhaustion or when participants failed to return three consecutive balls. Target accuracy was assessed during the final 2 min of the incremental test using a 40 × 40 cm target zone positioned in the deep forehand area, 10 cm from both the end line and sideline. A hit was recorded when a legal forehand topspin returns first bounced within the target zone or on its boundary. Returns landing outside the zone, into the net, or off the table, as well as missed balls, were recorded as unsuccessful. Accuracy was calculated as the number of target hits divided by the total number of balls delivered during the final 2 min × 100%. The same independent coach, blinded to group allocation, assessed all pre- and post-intervention tests in real time using identical criteria. Immediately after test termination, participants remained seated passively while heart rate was continuously recorded using a Polar H10 chest strap. HRR60 was calculated as the absolute difference between peak exercise heart rate and heart rate exactly 1 min after exercise (Buchheit et al., 2007). The outcomes derived from this protocol were total time to exhaustion, maximal ball-launching frequency, fatigued target accuracy, and HRR60. This protocol has been shown to reflect the near-maximal cardiovascular demands of competitive match play (Zagatto et al., 2016).

### 2.4 Training program

Both groups completed an 8-week court-based table tennis intervention. Certified coaches supervised five 90–120-min sessions per week. The training program progressed systematically over the intervention period. The initial weeks focused on technical consistency and familiarization with the training protocol. During the middle phase, training shifted toward sport-specific endurance, high-demand footwork, and sustained rally execution. During the final 2 weeks, intensity targets were maintained while overall training load was tapered to reduce fatigue before post-testing. Each session consisted of standardized training blocks. Each session began with a 10–15-min warm-up consisting of dynamic stretching and low-intensity rallying. This was followed by a 25–30-min technical block emphasizing serve-and-receive patterns and controlled stroke mechanics. Coaches then adjusted 20–30-min footwork and multiball blocks to match the assigned physiological intensity zone. Tactical match-play simulations lasted another 20–30 min and were followed by a brief cool-down. The experimental manipulation targeted physiological intensity distribution rather than table tennis stroke mechanics. Both groups performed comparable technical drills, multiball sequences, and match simulations, with no supplemental off-table endurance training. Coaches regulated physiological load by manipulating drill duration, ball-feeding frequency, movement radius, and recovery intervals according to each participant’s baseline CPET data. Zone 1 (Z1) was defined as heart rate below VT1. Zone 2 (Z2) was defined as heart rate between VT1 and VT2. Zone 3 (Z3) was defined as heart rate above VT2 (**Table 1**) (Stöggl and Sperlich, 2014).

**Table 1:** Overview of the 8-week training progression in the POL and THR groups.

Phase	Objective	POL emphasis	THR emphasis
Weeks 1-2	Familiarization and technique stability	Mostly Z1; brief introductory Z3 intervals; Z2 minimized	Z1 plus longer Z2 rally/multiball blocks; very little Z3
Weeks 3-6	Main loading phase	Target 80% Zone 1/5% Zone 2/15% Zone 3 distribution; 2–3 Z3 blocks weekly	Target 50% Zone 1/45% Zone 2/5% Zone 3 distribution; sustained Z2 drills emphasized
Weeks 7-8	Consolidation before post-testing	Distribution maintained; avoid unnecessary fatigue	Distribution maintained; avoid unnecessary fatigue

#### 2.4.1 Polarized training group

The POL intervention allocated approximately 80% of training time to Z1, 5% to Z2, and 15% to Z3. Z1 training emphasized low-intensity stroke mechanics, serve returns, and continuous multiball drills maintained below VT1. Z3 training consisted of short high-intensity intervals performed above VT2. These blocks included rapid multiball feeding, explosive footwork sequences, and aggressive attacking patterns. A standard Z3 interval consisted of 30–45 s of maximal effort followed by 60–90 s of passive recovery. Players completed 6–10 repetitions per block, with blocks separated by several minutes of Z1 active recovery. Z2 exposure occurred only incidentally during physiological transitions between low- and high-intensity work.

#### 2.4.2 Threshold training group

The THR protocol followed a threshold-based training model. Total training time was distributed as approximately 50% in Z1, 45% in Z2, and 5% in Z3. Z2 training formed the core of this intervention and consisted of sustained moderate-intensity table tennis drills. Players performed prolonged multiball sets, extended rally sequences, and match-play simulations while maintaining heart rate between VT1 and VT2. Continuous work periods of 4–8 min alternated with 2–3-min recovery periods. These blocks were repeated up to six times per session. Brief Z3 efforts occurred only incidentally during the natural flow of match-play simulations.

#### 2.4.3 Training load monitoring

Internal load was monitored using daily session rating of perceived exertion (sRPE) and continuous heart rate telemetry. Weekly time-in-zone averages were calculated to verify adherence to the assigned training protocol. Time in zone was computed from the continuous heart rate recording of each complete session and expressed as a percentage of total session duration. These values was not corrected for the lag in heart rate response to changes in work rate, because no standard correction procedure is established for this method and adjusted values would not be comparable with those reported elsewhere in the training intensity distribution literature. In the POL protocol, heart rate continued to rise briefly after each 30 to 45 s repetition ended, and the 60 to 90 s of passive recovery between repetitions was shorter than the time needed for heart rate to fall below VT2 once the first repetitions of a block had been completed. Time accumulated during these blocks was therefore recorded predominantly in Zone 3 rather than displaced into Zone 1 or Zone 2. The Zone 3 percentage in **Table 2** represents the time for which heart rate exceeded VT2 and not the time spent performing maximal work, which corresponded to one third of total interval time given the prescribed 1:2 ratio of work to recovery. Thirty minutes after each session, participants reported sRPE using the Borg CR10 scale. Total internal load was calculated by multiplying the sRPE value by session duration. These metrics were reviewed weekly by the coaching staff. Ball-feeding frequency and recovery ratios were then adjusted to ensure that total internal workload remained matched between groups.

**Table 2:** Training adherence, internal load, and monitored intensity distribution during the 8-week intervention.

Variable	POL group	THR group
Session duration (min)	105.4 ± 8.2	107.1 ± 7.5
Mean training HR (bpm)	142 ± 6	145 ± 5
Weekly cumulative sRPE load (AU)	3245 ± 195	3312 ± 210
Time in Z1 (%)	78.5 ± 3.2	52.4 ± 4.1
Time in Z2 (%)	5.7 ± 1.5	41.1 ± 3.6
Time in Z3 (%)	15.8 ± 2.5	6.5 ± 1.8

### 2.5 Statistical analysis

Data are presented as mean ± standard deviation (M ± SD). Statistical analyses were performed using SPSS Statistics software (version 28.0; IBM Corp., Armonk, NY, USA), with the alpha level set at 0.05 for all tests. Normality was assessed using the Shapiro–Wilk test before inferential analyses. Intervention effects were examined using two-way mixed-design analyses of variance (ANOVAs), with group (POL vs. THR) as the between-subjects factor and time (pre vs. post) as the within-subjects factor. When a significant group × time interaction was detected, Bonferroni-corrected *post hoc* comparisons were performed. Effect sizes were reported as partial eta squared (ηp^2^), with values of 0.01, 0.06, and 0.14 interpreted as small, medium, and large, respectively. Cohen’s d was also calculated for within-group pre-to-post changes. Baseline comparability between groups was assessed using independent-samples t-tests (Hopkins et al., 2009). For outcomes showing appreciable baseline imbalance, supplementary ANCOVA was performed. For outcomes showing apparent heterogeneity in individual responses, participants were classified according to whether their post-intervention values increased or decreased relative to baseline. Fisher’s exact test was used to compare the proportions of participants showing decreases between groups.

## 3 Results

### 3.1 Baseline characteristics

Baseline characteristics are presented descriptively by group in **Table 2**; no meaningful differences were apparent between groups. Training load monitoring was consistent with similar internal workload accumulation throughout the 8-week intervention. Session duration, mean training heart rate, and weekly cumulative sRPE load were similar between groups. Time-in-zone analysis confirmed adherence to the prescribed intensity distributions, with the POL group completing most training time in Zone 1 and the THR group accumulating substantially greater time in Zone 2 (**Table 2**).

### 3.2 Cardiorespiratory fitness adaptations

**Figure 1:**
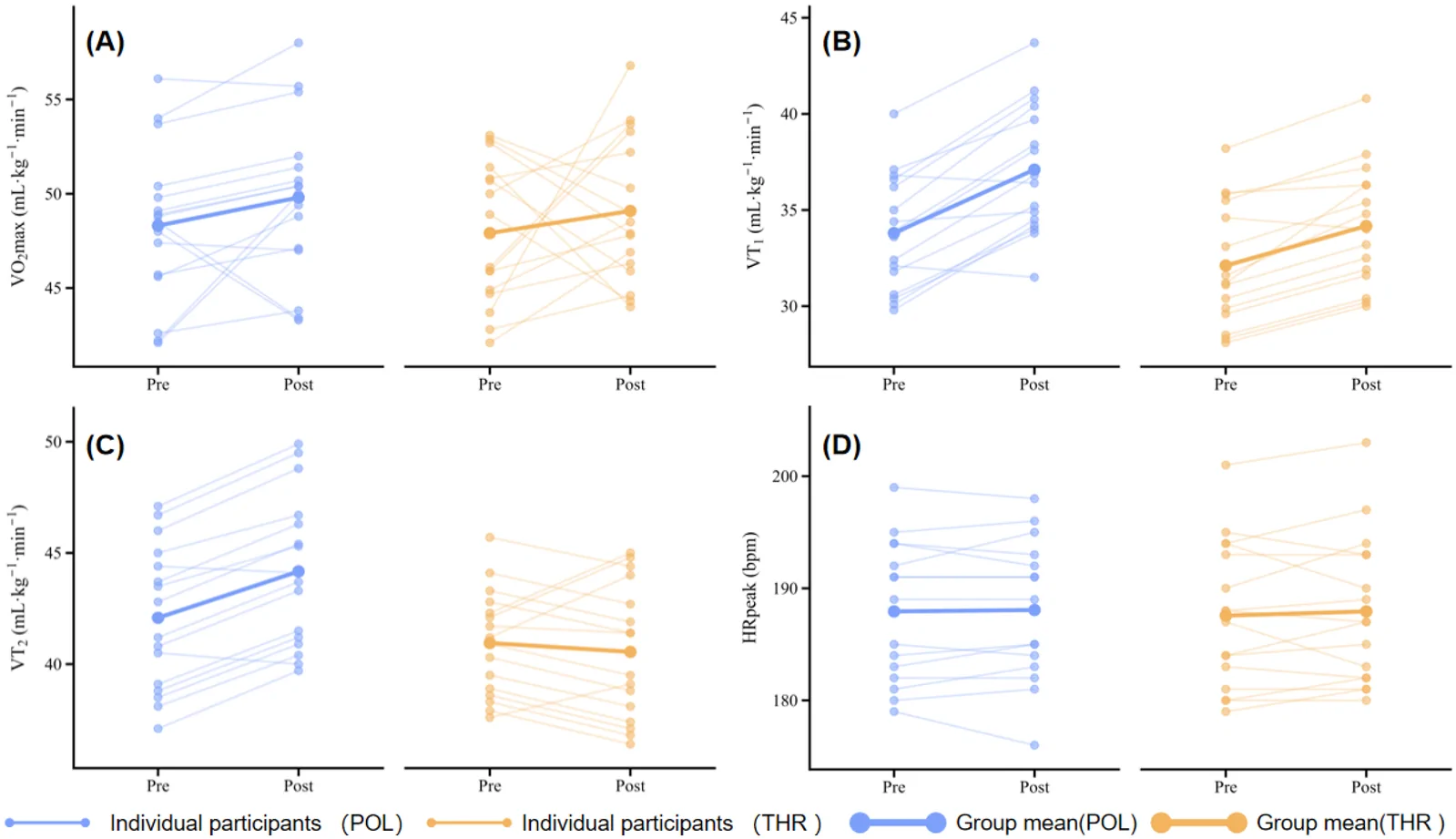
Individual and group mean changes in cardiorespiratory fitness parameters from pre- to post-intervention in the polarized training (POL) and threshold training (THR) groups. Panels display pre- to post-intervention changes for: **(A)** maximal oxygen uptake (VO_2_max, mL·kg^-1^·min^-1^); **(B)** first ventilatory threshold (VT_1_, mL·kg^-1^·min^-1^); **(C)** second ventilatory threshold (VT_2_, mL·kg^-1^·min^-1^); and **(D)** peak heart rate (HRpeak, bpm).

An exploratory analysis of individual changes showed that VT2 decreased from baseline in 12 of 16 participants in the THR group, compared with 2 of 16 participants in the POL group (Fisher’s exact p = 0.001). In contrast, target accuracy decreased in 6 of 16 participants in each group (p = 1.000). VT1 showed a statistically significant Time × Group interaction (F (1, 30) = 5.08, p = 0.03, ηp^2^ = 0.145; **Table 3**, **Figure 1B**). VT1 increased from pre- to post-test in both groups (POL: p< 0.001; THR: p< 0.001); The relative improvement was greater in the POL group (9.80% vs. 6.40%), with a significant between-group difference at post-test (p = 0.02). VT2 also showed a statistically significant Time × Group interaction (F (1, 30) = 24.52, p< 0.001, ηp^2^ = 0.450; **Table 3**, **Figure 1C**), with an increase in the POL group (+5.00%, p< 0.001; n = 16) and no statistical evidence of change in the THR group (−1.00%, p = 0.270; n = 16). No Time × Group interactions were found for absolute $\dot{V}O_{2\max}$ (F (1, 30) = 0.05, p = 0.821, ηp^2^ = 0.002; **Table 3**, **Figure 1A**) or peak heart rate (F (1, 30) = 0.13, p = 0.726, ηp^2^ = 0.004; **Table 3**, **Figure 1D**).

**Table 3:** Pre- and post-intervention changes in physiological and table tennis-specific performance variables in the POL and THR groups.

Variable	POL group				THR group				ANOVA P values (η_p_^2^) (η_p_^2^category)		
	Pre	Post	Δ (%)	ES	Pre	Post	Δ (%)	ES	Time×group	Time	Group
VO_2_max (mL·kg^−1^·min^−1^)	48.31 ± 4.11	49.80 ± 4.30	3.10	0.35	47.91 ± 3.80	49.09 ± 3.90	2.5	0.31	0.82 (0.002) Small	0.07 (0.108) Medium	0.66 (0.007) Small
VT1 (mL·kg^−1^·min^−1^)	33.80 ± 2.99	37.10 ± 3.37*	9.80	1.04	32.11 ± 3.09	34.17 ± 3.07*#	6.40	0.67	0.03 (0.145) Large	<0.01 (0.758) Large	0.04 (0.134) Medium
VT2 (mL·kg^−1^·min^−1^)	42.08 ± 3.26	44.17 ± 3.42*	5.00	0.63	40.95 ± 2.38	40.55 ± 3.03#	-1.00	-0.15	<0.01 (0.45) Large	<0.01 (0.273) Large	0.03 (0.146) Large
HRpeak (bpm)	187.94 ± 6.09	188.06 ± 6.12	0.10	0.02	187.56 ± 6.49	187.94 ± 6.67	0.20	0.06	0.73 (0.004) Small	0.48 (0.016) Small	0.91 (0.000) Small
TTE-specific (s)	596.36 ± 39.92	612.69 ± 46.14*	2.70	0.38	570.50 ± 46.31	571.94 ± 51.74#	0.30	0.03	0.049 (0.124) Medium	0.02 (0.167) Large	0.045 (0.127) Medium
Max ball frequency (balls/min)	89.98 ± 7.01	90.42 ± 7.36	0.50	0.06	88.99 ± 5.99	89.54 ± 6.95	0.60	0.08	0.91 (0.000) Small	0.32 (0.033) Small	0.70 (0.005) Small
Target accuracy rate (%)	62.65 ± 7.37	67.53 ± 7.26*	7.80	0.67	59.65 ± 7.43	60.55 ± 5.21#	1.50	0.14	0.04 (0.132) Medium	<0.01 (0.253) Medium	0.03 (0.141) Large
HRR60 (bpm)	28.94 ± 4.81	32.31 ± 5.34*	11.70	0.66	26.94 ± 4.25	26.94 ± 4.81#	0.00	0	0.04 (0.133) Medium	0.040 (0.133) Medium	0.02 (0.166) Large

Values are presented as mean ± SD. Δ (%) indicates the percentage change from pre- to post-intervention. POL, polarized training; THR, traditional threshold training; ES, Effect Size; VO_2_max, maximal oxygen uptake; VT1, first ventilatory threshold; VT2, second ventilatory threshold; HRpeak, peak heart rate; TTE-specific, table tennis-specific time to exhaustion; HRR60, heart rate recovery at 60 s; ηp^2^, partial eta squared. ANOVA p values and ηp^2^ were obtained from two-way repeated-measures ANOVA. Effect-size categories for ηp^2^ were defined as small (<0.06), medium (0.06–0.139), and large (≥0.14). p< 0.05 indicates a significant difference from pre-intervention within the same group. *p< 0.05 indicates a significant pre-to-post change within the same group. # indicates a significant between-group difference at post-test after Bonferroni adjustment: VT1, p = 0.015; VT2, p = 0.004; TTE-specific, p = 0.025; target accuracy, p = 0.004; and HRR60, p = 0.005.

### 3.3 Table tennis-specific performance and post-exercise heart-rate recovery

**Figure 2:**
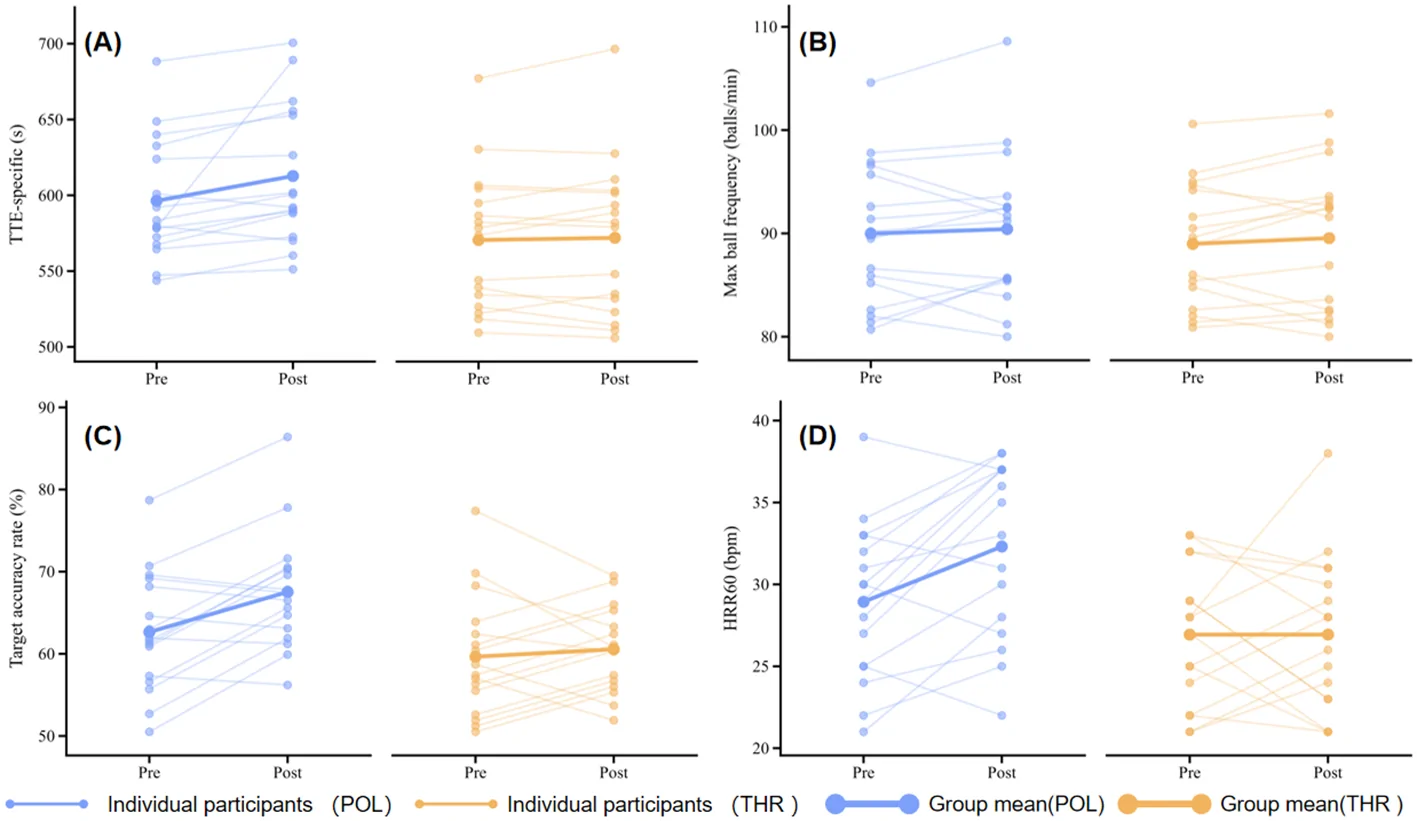
Individual and group mean changes in table tennis-specific performance and autonomic recovery from pre- to post-intervention in the polarized training (POL) and threshold training (THR) groups. Panels display pre- to post-intervention changes for: **(A)** sport-specific time to exhaustion (TTE-specific, s); **(B)** maximum ball frequency (balls/min); **(C)** target accuracy rate (%); and **(D)** heart rate recovery at 60 seconds post-exercise (HRR60, bpm).

Sport-specific TTE showed a significant Time × Group interaction (F (1, 30) = 4.23, p = 0.049, ηp^2^ = 0.124; **Table 3**, **Figure 2A**). The POL group showed a significant pre-to-post increase in TTE (+2.70%, p = 0.003), whereas no statistical evidence of pre-to-post TTE change was detected in the THR group (p = 0.781). However, in the supplementary ANCOVA, the baseline-adjusted group effect did not reach statistical significance (adjusted mean difference = 14.10 s, 95% CI [−1.63, 29.84], F(1, 29) = 3.36, p = 0.077, ηp^2^ = 0.104). Target accuracy during the terminal stage of the incremental protocol also showed a significant Time × Group interaction (F (1, 30) = 4.56, p = 0.041, ηp^2^ = 0.132; **Table 3**, **Figure 2C**). The POL group showed a significant increase in target accuracy (+7.80%, p = 0.001; n = 16), while no statistical evidence of pre-to-post change was detected in the THR group (p = 0.500; n = 16). No Time × Group interaction was observed for maximum ball frequency (F (1, 30) = 0.01, p = 0.909, ηp^2^< 0.001; **Table 3**, **Figure 2B**). HRR60 showed a significant Time × Group interaction (F (1, 30) = 4.62, p = 0.040, ηp^2^ = 0.133; **Table 3**, **Figure 2D**). The POL group showed an 11.70% increase in HRR60, consistent with faster post-exercise heart-rate recovery (p = 0.005; n = 16), whereas no statistical evidence of pre-to-post change was detected in the THR group (p = 1.000; n = 16). This divergent response resulted in a between-group difference at post-test (p = 0.005).

## 4 Discussion

To our knowledge, this is among the first randomized controlled trials to compare a POL intensity distribution with a THR model for performance attributes in table tennis players. After an 8-week intervention with broadly comparable accumulated internal load, the POL group showed significantly greater improvements in submaximal physiological markers (VT1 and VT2), sport-specific endurance, fatigue-induced technical stability, and autonomic recovery, as reflected by HRR60. These findings question the conventional emphasis on high volumes of moderate-intensity training in skill-based intermittent sports. They also suggest that the distribution of training intensity may be as important as total workload in determining adaptive responses.

A central finding of this study was that the POL group showed greater improvements in ventilatory thresholds. The significant improvements in both VT1 and VT2 are particularly relevant to table tennis, which is characterized by repeated high-intensity rallies interspersed with brief recovery periods (Pion et al., 2015). An elevated VT1 reflects improved submaximal aerobic function and may support recovery between points and delay fatigue during prolonged matches. Previous studies have associated high-volume low-intensity training with mitochondrial and capillary adaptations; however, these mechanisms were not directly assessed in the present study and should therefore be regarded only as possible explanations (Zapata-Lamana et al., 2018). In addition, the improvement in VT2 in the POL group, together with the lack of meaningful change in the THR group, suggests the efficacy of high-intensity Zone 3 stimuli. These high-intensity bouts may challenge lactate transport and buffering systems, thereby increasing the highest sustainable exercise intensity before substantial metabolic disturbance occurs (Chiang et al., 2023). In contrast, the extensive time spent in Zone 2 by the THR group may have provided an insufficient stimulus for further VT2 adaptation. This pattern may reflect high physiological strain without a proportional adaptive signal, a phenomenon described in the endurance training literature (Stöggl and Sperlich, 2014).

The Zone 3 and Zone 2 exposures to which these responses are attributed depend on how intensity distribution was quantified. Intensity distribution can be quantified either by the time accumulated in each zone or by classifying whole sessions according to their primary intended stimulus, and the two approaches applied to the same training data yield different distributions (Sylta et al., 2014). The session-goal method assigns each session in full to its target zone, so that a session built around high-intensity intervals contributes its warm-up, recovery, and cool-down to Zone 3, which generally raises the reported share of high-intensity training relative to the time-in-zone method. This method could not be applied in the present study because both groups completed sessions of identical structure and the intensity manipulation was made within sessions rather than between them. The distributions reported here are therefore most directly comparable with studies that used time in zone, and should be read as descriptions of the cardiovascular demand accumulated across the whole session rather than of the intensity prescribed for each drill.

Although submaximal fitness markers differed between groups, no significant group-by-time interaction was observed for $\dot{V}O_{2\max}$. This finding is consistent with studies comparing POL with other training models that reported similar improvements in maximal aerobic power, particularly after short-term interventions (Rosenblat et al., 2019). However, other meta-analytic evidence suggests that POL may be more effective for improving $\dot{V}O_{2\max}$, particularly in highly trained athletes and during shorter interventions (Silva Oliveira et al., 2024). Therefore, the 8-week intervention may have been insufficient to produce differential $\dot{V}O_{2\max}$ adaptations in this cohort of trained players. More importantly, in an intermittent sport such as table tennis, $\dot{V}O_{2\max}$ may be less predictive of performance than fatigue resistance and rapid recovery, which may be better reflected by submaximal thresholds and sport-specific endurance tests (Rivera-Köfler et al., 2025). CRF is a multidimensional construct, and its components may contribute differently to performance across sporting disciplines (Kodama et al., 2009; Ross et al., 2016). Our results suggest that, in table tennis, enhancing the aerobic foundation reflected by VT1 and the capacity for sustainable high-intensity work reflected by VT2 may be more relevant than increasing absolute $\dot{V}O_{2\max}$.

The strongest evidence for the efficacy of the POL model in this study was the translation of physiological adaptations into sport-specific performance gains. The greater improvement in target accuracy under fatigue, together with the favorable TTE response, suggests a possible link between metabolic conditioning and technical execution; however, the TTE finding should be interpreted cautiously because the baseline-adjusted group effect was not statistically significant. The ability to maintain fine motor control and shot precision under physiological stress is a key feature of high-level performance. The enhanced aerobic base in the POL group may have supported better metabolic homeostasis during the incremental test, thereby reducing the accumulation of fatigue-related metabolites that could impair neuromuscular function and cognitive processing (Oberlin et al., 2025). This preservation of technical skill under standardized fatigue may represent a potentially relevant performance advantage that was not observed after the THR program. At the individual level, observed decreases in VT2 were more frequent in the THR group, whereas decreases in target accuracy occurred equally in both groups. This finding provides additional context for the group-level VT2 response; however, because no measurement-error threshold was available, it does not establish true non-response or confirm that the observed decreases resulted from residual neuromuscular fatigue.

Furthermore, the faster post-exercise HRR60 observed in the POL group indicates faster heart-rate recovery and may reflect improved autonomic regulation. HRR is commonly used as an indirect indicator of parasympathetic reactivation, which is important for recovery between points and matches (Pathak et al., 2015). Previous research suggests that high volume of low-intensity training in the POL model may have promoted greater parasympathetic modulation, whereas the sustained moderate-intensity stress in the THR model may have prolonged sympathetic activation. However, HRV and other direct measures of autonomic function were not assessed in the present study; therefore, these mechanisms remain speculative. This faster heart-rate recovery may allow athletes to begin subsequent points in a more recovered state, thereby supporting explosive actions and complex decision-making. This finding supports our initial hypothesis and highlights an important but often overlooked benefit of the polarized approach: improved recovery capacity, which may be as important as the training stimulus itself.

From a practical perspective, these findings may provide useful guidance for coaches working in table tennis and other high-skill intermittent sports. The results suggest that a training paradigm heavily reliant on moderate-intensity Zone 2 work may be less effective for developing the physiological qualities required for performance in these sports. In contrast, a polarized approach combines a large volume of low-intensity, technically focused practice with a smaller volume of targeted high-intensity sport-specific drills and may represent a more effective strategy. This model may support physiological adaptation while allowing more training time for skill refinement in a relatively non-fatigued state. High-intensity sessions can then provide a targeted stimulus to challenge sport-specific physical capacities. By reducing prolonged exposure to moderate-intensity work, the large Zone 1 component may limit cumulative sympathetic strain and provide greater opportunity for parasympathetic recovery between sessions. This balance between training stress and recovery may help athletes tolerate high training volumes while reducing the risk of non-functional overreaching or burnout (Filipas et al., 2022).

Several limitations should be acknowledged. First, the sample consisted exclusively of male athletes. Therefore, the findings cannot be directly generalized to female players, who may show different training adaptations because of hormonal fluctuations and other physiological differences (Recacha-Ponce et al., 2025), and the optimal training intensity distribution may also differ in international-level athletes. Longer interventions spanning a competitive season are therefore needed. Second, although internal training loads were comparable, external load was not directly quantified. Moreover, the on-court training zones were derived from treadmill-based heart-rate thresholds without a mode-specific correction factor and may not have precisely reflected the metabolic intensity of the table tennis drills. Intensity distribution was quantified by time in zone alone, and heart rate is an indirect index of intensity whose response lags behind changes in work rate during repetitions as short as those used in the POL protocol. Third, the test–retest reliability and typical error of the target-accuracy measure were not established, so the observed change should be interpreted cautiously. Finally, the fixed ball placement, single stroke type, and predictable feeding pattern did not fully reproduce the multidirectional and unpredictable demands of match play. Accordingly, the technical findings reflect performance under standardized fatigue conditions rather than direct evidence of competitive performance. Future studies should quantify external load, establish the reliability of target accuracy, and use more ecologically valid protocols with biomechanical analyses.

## 5 Conclusion

This study suggests that an 8-week polarized training program may be more effective than a traditional threshold-based program for improving submaximal cardiorespiratory fitness, sport-specific endurance, fatigue-induced technical stability, and autonomic recovery in competitive male table tennis players. The strategic emphasis on low- and high-intensity training, combined with reduced moderate-intensity work, may promote a physiological profile well suited to the demands of competitive table tennis. These findings suggest that coaches and athletes in skill-based intermittent sports may consider adopting a polarized training intensity distribution to improve performance.

## Data Availability

The raw data supporting the conclusions of this article will be made available by the authors, without undue reservation.
